# Supplementary material for: Hippocampal-prefrontal connectivity relates to inter-individual differences and training gains in distinguishing similar memories
Source: Commun Biol. 2025 Dec 28;9:129. doi: 10.1038/s42003-025-09408-7 (PMC12855975; doi:10.1038/s42003-025-09408-7)
Supplement: Supplementary file 2 — Supplementary Information [file 42003_2025_9408_MOESM2_ESM.pdf]

Supplementary information

Hippocampal-prefrontal connectivity relates to inter-individual differences and training gains in distinguishing similar memories

Panagiotis Iliopoulos[1,2*]+, Jeremie Güsten[1*], Eóin Molloy[2,3,4], Radoslaw Martin Cichy[5], Friedrich Krohn[1,2], Anne Maass[2] & Emrah Düzel[1,2]

1: Institute of Cognitive Neurology and Dementia Research, Otto-von-Guericke University, Magdeburg, Germany

2: German Center for Neurodegenerative Diseases (DZNE), Magdeburg, Germany

3: Division of Nuclear Medicine, Department of Radiology & Nuclear Medicine, Faculty of Medicine, Otto von Guericke University Magdeburg, Magdeburg, Germany

4: AICURA medical GmbH, Colditzstraße 34/36, 16A, 12099 Berlin, Germany

5: Department of Education and Psychology, Freie Universität Berlin, Berlin, Germany

*authors contributed equally to this work.

+ contact: panagiotis.iliopoulos@med.ovgu.de

Results

S2.1 Behavioral data at baseline

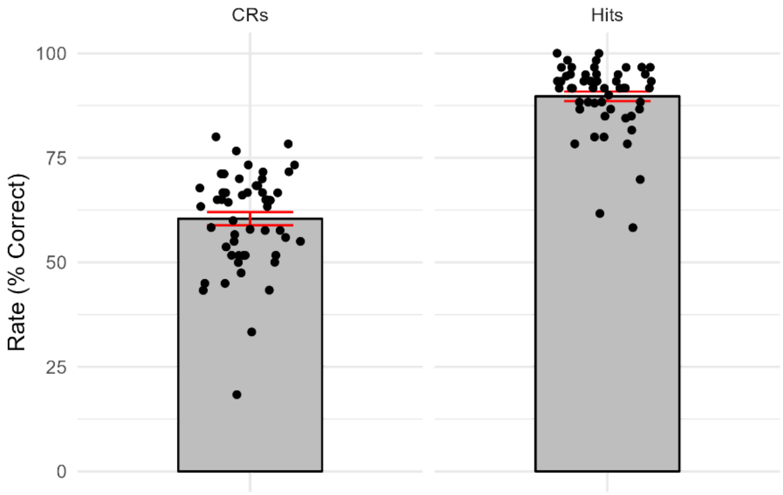

Figure S1. Behavioral performance at baseline (pre-training) in the object-scene MD task. The performance (percent correct) is plotted separately for the correct repeats (hits) and the correct lure

trials (CRs – correct rejections) for the whole sample (*n=54*). All data points correspond to the baseline (pre-training) time point. The error bars show the standard error of the mean.

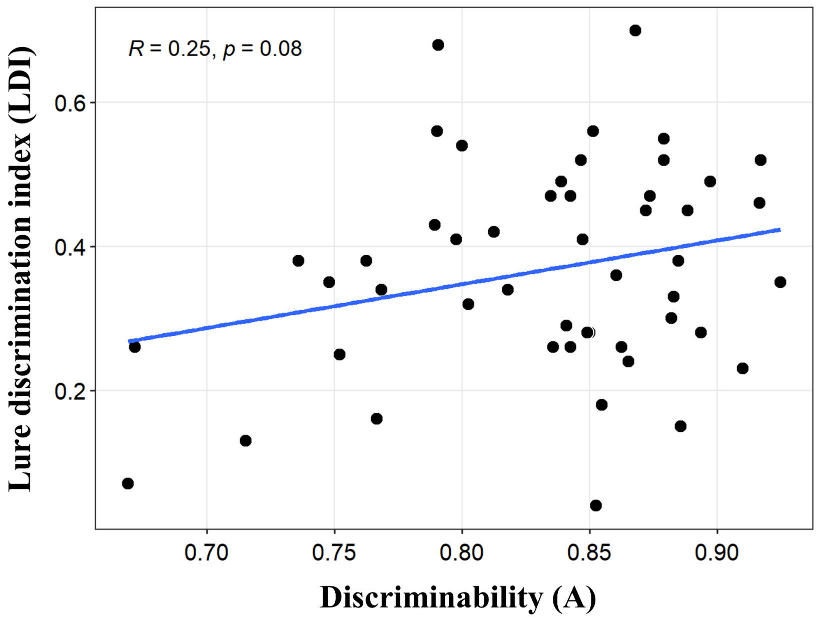

Figure S2. Correlation between baseline (pre-training) performance on the two different mnemonic discrimination tasks. The X axis represents performance on the object-scene MD task (discriminability A)**,** while the Y axis represents performance on the MST task (LDI index) for the whole sample (*n=54*). Both measures are bias-corrected and derived from their respective MD tasks. All data points correspond to the baseline (pre-training) time point. *R* = correlation index (Pearson r), *p* = p-value.

Table S1

Multivariate regression model of the composite connectivity score (hippocampal-PFC) effect on the combined behavioral measures

IV	Pillai test	F	$DF_{n,d}$	*p*
Composite Connectivity Score	0.346	3.18	6, 36	**.013**
Age	0.194	1.44	6, 36	.226
Sex	0.176	1.29	6, 36	.289

Note. Multivariate regression model. The LD composite connectivity score in the hippocampal-PFC connections is the main predictor variable (age, sex: covariates of no interest). The combined behavioral measures (described in Table S2) are the dependent variables. IV: independent variable. Values in bold indicate $P < 0.05$.

Table S2

Effects of composite connectivity score (hippocampal-PFC) on memory behavioral measures

Task	DV	b	SE	*t*	*p*
MST	LDI	-0.056	0.028	-2.01	.0516
MST	Corrected Hit Rate	-0.055	0.024	-2.25	.0298
ORR	Correct Retrieval	-2.324	1.738	-1.34	.1886
ORR	Retrieval: Internal Errors	0.024	1.000	0.02	.981
ROCF	Delay Recall	0.440	0.769	0.57	.5707
Verbal Learning	Encoding vs Delay-recall Score	-0.127	0.360	-0.35	.727

Note. Linear regression effect of the LD Composite connectivity score in the hippocampal-PFC connections on separate behavioral memory measures and tasks. Model included age and sex as covariates of no interest. MST: mnemonic similarity task, Verbal Learning (word list learning), ORR: object-in-room recall task, ROCF: Rey-Osterrieth Complex Figure, DV: dependent variable. LDI: lure discrimination index, Corrected Hit Rate (hits minus false alarms). The hippocampal-PFC composite connectivity score shows a negative relationship with the LDI and Corrected Hit Rate measures in the MST task.

S2.2 ANOVA cognitive training effects

Table S3

ANOVA cognitive training results for discriminability index A.

DV	Effect	Condition	Mean	SE	$DF_{n,d}$	F	*p*	η^2_G	η^2_p
A	Group	Control	0.851	0.009	1,49	4.94	.031	0.070	0.092
		Training	0.880	0.009					
	Time	Pre	0.839	0.008	1,49	52.59	< .001	0.210	0.518
		Post	0.892	0.006					
	Group x Time	Control / Pre	0.836	0.011	1,49	9.34	.004	0.045	0.160
		Training / Pre	0.842	0.012					
		Control / Post	0.867	0.009					
		Training / Post	0.917	0.009					

Note. Cognitive training results using ANOVA on A discriminability values with factors "Group" (control, training; between-subject) and Time (pre, post-training; within-subject). Group x Time interaction and main effects are shown. Given are mean values and standard error of the mean (SE), degrees of freedom for the numerator (DFn) and denominator (DFd), p-values (p), and effect size (η^2_g, η^2_p). P-values < 0.05 are considered as significant. DV= dependent variable.

Table S4

ANOVA cognitive training results for the LOC-OP LD connectivity

Effect	Condition	Mean	SE	$DF_{n,d}$	F	p	p-FDR	η^2_G	η^2_p
Group	Control	-1.92	0.666	1,49	0.02	.902	.960	<.001	<.001
	Training	-2.04	0.697						
Time	Pre	-4.01	0.675	1,49	17.91	< .001	< .001	0.154	0.268
	Post	0.06	0.689						
Group x Time	Control / Pre	-2.44	0.932	1,49	10.00	.003	.016	0.092	0.170
	Training / Pre	-5.59	0.975						
	Control / Post	-1.40	0.951						
	Training / Post	1.52	0.994						

Note. Cognitive training ANOVA results for the LOC-OP LD connectivity values (dependent variable) with factors "Group" (control, training; between-subject) and Time (pre, post-training; within-subject). Group x Time interaction and main effects are shown. Given are mean values and standard error of the mean (SE), degrees of freedom for the numerator (DFn) and denominator (DFd), p-values (p), and effect size (η^2_g, η^2_p). Within cluster p-FDR correction was applied to the p-values, p-FDR < 0.05 are considered significant.

Table S5
ANOVA cognitive training results for connectivity values in cluster 1

Connection	Condition	Mean	SE	$DF_{n,d}$	F	p	p-FDR	η^2_G	η^2_p
LOC-OP	Control / Pre	-2.44	0.93	1,49	10.00	**.003**	**.016**	0.092	0.170
	Training / Pre	-5.59	0.98						
	Control / Post	-1.40	0.95						
	Training / Post	1.52	0.99						
OP-LOC	Control / Pre	-1.40	0.39	1,49	0.19	.663	.796	0.002	0.004
	Training / Pre	-1.12	0.41						
	Control / Post	-0.57	0.44						
	Training / Post	0.07	0.46						
PRC-OP	Control / Pre	-1.67	0.75	1,49	0.41	.525	.787	0.003	0.008
	Training / Pre	-1.31	0.78						
	Control / Post	0.29	0.68						
	Training / Post	-0.21	0.71						
OP-EC	Control / Pre	-1.69	0.66	1,49	0.44	.509	.787	0.004	0.009
	Training / Pre	-1.05	0.69						
	Control / Post	0.54	0.78						
	Training / Post	0.20	0.81						
OP-PRC	Control / Pre	-2.30	0.88	1,49	2.74	.104	.313	0.021	0.053
	Training / Pre	-0.33	0.92						
	Control / Post	0.61	0.85						
	Training / Post	0.03	0.89						
EC-OP	Control / Pre	-1.42	0.83	1,49	0.01	.927	.927	<.001	<.001
	Training / Pre	-1.53	0.87						
	Control / Post	0.48	0.75						
	Training / Post	0.50	0.79						

Note. ANOVA results examining the effect of cognitive training (Group x Time interaction) on connectivity for each individual connection (factors 'Group': control, training; between-subject. 'Time': pre-, post-training; within-subject). Given are mean values and standard error of the mean (SE), degrees of freedom for the numerator (DFn) and denominator (DFd), p-values (p), and effect size (η^2_g, η^2_p). Within cluster p-FDR correction was applied to the p-values. P-FDR < 0.05 are considered as significant. Values in bold indicate P < 0.05.

Table S6.

ANOVA cognitive training results for connectivity values in cluster 2

Connection	Condition	Mean	SE	$DF_{n,d}$	F	p	p-FDR	η^2_G	η^2_p
LOC-IFG tri	Control / Pre	2.01	0.794	1,49	0.14	.710	.911	0.002	0.003
	Training / Pre	2.66	0.831						
	Control / Post	0.23	0.744						
	Training / Post	0.20	0.779						
LOC-SFG	Control / Pre	1.61	0.746	1,49	0.01	.911	.911	<.001	<.001
	Training / Pre	1.60	0.780						
	Control / Post	0.22	0.559						
	Training / Post	0.05	0.584						
LOC-IFG oper	Control / Pre	0.56	0.758	1,49	1.69	.200	.534	0.020	0.033
	Training / Pre	2.49	0.793						
	Control / Post	-0.43	0.678						
	Training / Post	-0.57	0.709						
IFG tri-LOC	Control / Pre	1.44	0.711	1,49	0.02	.903	.911	<.001	<.001
	Training / Pre	1.77	0.744						
	Control / Post	0.55	0.723						
	Training / Post	1.08	0.757						
SFG-LOC	Control / Pre	1.26	1.166	1,49	0.59	.445	.890	0.005	0.012
	Training / Pre	2.44	1.220						
	Control / Post	1.16	0.985						
	Training / Post	0.78	1.030						
LOC-HIPP	Control / Pre	1.13	0.901	1,49	2.15	.149	.534	0.023	0.042
	Training / Pre	1.57	0.942						
	Control / Post	1.34	0.782						
	Training / Post	-0.84	0.818						
IFG tri-EC	Control / Pre	1.25	1.283	1,49	0.07	.786	.911	<.001	0.002
	Training / Pre	1.54	1.343						
	Control / Post	0.71	0.908						
	Training / Post	0.34	0.950						
PRC-IFG tri	Control / Pre	0.24	0.470	1,49	4.03	**.050**	.401	0.039	0.076
	Training / Pre	1.31	0.491						
	Control / Post	0.56	0.440						
	Training / Post	-0.23	0.461						

Note. ANOVA results examining the effect of cognitive training (Group x Time interaction) on connectivity for each individual connection (factors ‘Group’: control, training; between-subject. ‘Time’: pre-, post-training; within-subject). Given are mean values and standard error of the mean (SE), degrees of freedom for the numerator (DFn) and denominator (DFd), p-values (p), and effect size (η^2_g, η^2_p). Within cluster p-FDR correction was applied to the p-values. P-FDR < 0.05 are considered as significant. Values in bold indicate P = 0.05

Table S7.

ANOVA cognitive training results for connectivity values in cluster 3

Connection	Condition	Mean	SE	$DF_{n,d}$	F	p	p-FDR	η^2_G	η^2_p
SFG-HPC	Control / Pre	2.45	0.880	1,49	1.09	.301	.967	0.011	0.022
	Training / Pre	2.80	0.921						
	Control / Post	1.07	1.154						
	Training / Post	-0.73	1.207						
IFG oper-HPC	Control / Pre	1.61	0.729	1,49	0.01	.921	.967	<.001	<.001
	Training / Pre	1.29	0.762						
	Control / Post	0.09	0.829						
	Training / Post	-0.07	0.868						
HPC-IFG oper	Control / Pre	1.86	0.897	1,49	0.01	.925	.967	<.001	<.001
	Training / Pre	1.44	0.938						
	Control / Post	-0.33	0.850						
	Training / Post	-0.59	0.890						
IFG tri-HIPP	Control / Pre	1.59	0.878	1,49	0	.967	.967	<.001	<.001
	Training / Pre	1.79	0.919						
	Control / Post	-0.29	0.784						
	Training / Post	-0.16	0.820						
HPC-SFG	Control / Pre	1.54	0.669	1,49	0.12	.733	.967	<.001	0.002
	Training / Pre	0.83	0.699						
	Control / Post	0.10	0.547						
	Training / Post	-0.99	0.572						
HPC-IFG tri	Control / Pre	1.52	1.142	1,49	0.06	.802	.967	<.001	0.001
	Training / Pre	1.11	1.195						
	Control / Post	-0.31	0.859						
	Training / Post	-1.21	0.898						
IFG tri-IFG oper	Control / Pre	1.14	0.664	1,49	0.57	.454	.967	0.006	0.012
	Training / Pre	0.54	0.695						
	Control / Post	-0.39	0.528						
	Training / Post	-0.02	0.552						

Note. ANOVA results examining the effect of cognitive training (Group x Time interaction) on connectivity for each individual connection (factors 'Group': control, training; between-subject. 'Time': pre-, post-training; within-subject). Given are mean values and standard error of the mean (SE), degrees of freedom for the numerator (DFn) and denominator (DFd), p-values (p), and effect size ($\eta^2_{g,}$ η^2_p). Within cluster p-FDR correction was applied to the p-values. P-FDR < 0.05 are considered as significant.

Table S8.

ANOVA cognitive training results for the LD HIPP-PFC composite connectivity

Effect	Condition	Mean	SE	$DF_{n,d}$	F	p	η^2_G	η^2_p
Group	Control	0.06	0.143	1,49	0.38	.542	0.004	0.008
	Training	-0.06	0.149					
Time	Pre	-0.02	0.144	1,49	0.05	.827	<.001	<.001
	Post	0.02	0.140					
Group x Time	Control / Pre	0.01	0.199	1,49	0.11	.739	0.001	0.002
	Training / Pre	-0.05	0.208					
	Control / Post	0.12	0.193					
	Training / Post	-0.07	0.202					

Note. Cognitive training ANOVA results for the HIPP-PFC LD composite connectivity (z-scored, dependent variable) with factors "Group" (control, training; between-subject) and Time (pre, post-training; within-subject). Group x Time interaction and main effects are shown. Given are mean values and standard error of the mean (SE), degrees of freedom for the numerator (DFn) and denominator (DFd), p-values (p), and effect size (η^2_g, η^2_p).

S2.3 Baseline connectivity-performance models

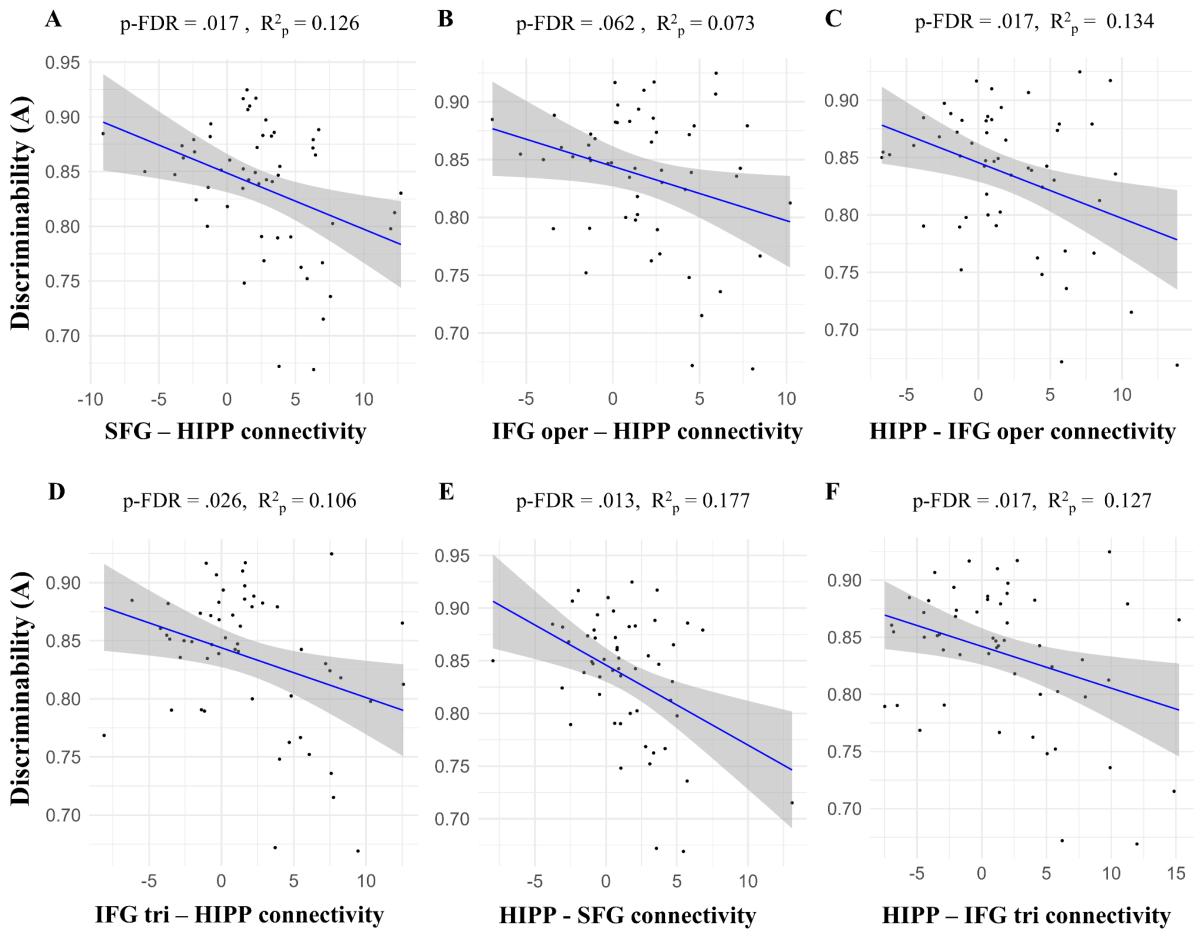

Figure S3. **Higher connectivity in the hippocampal-PFC connections identified in cluster 3 is associated with poorer MD performance**. Brain connectivity-behavior linear regression models fitted per each hippocampal-PFC connection identified in cluster 3 (ie: 6 out of the 7 in total connections in this cluster) . The X axis depicts the independent variable: LD connectivity values (gPPI) in a certain connection (seed ROI – target ROI). The Y axis shows the dependent variable: A discriminability. The p-FDR corrected and partial R square values are shown. FDR-correction was applied to all the connections within this cluster. In all connections depicted (A-F) we find a negative link with lower connectivity being associated with higher memory performance.

HIPP: hippocampus**,** **SFG**: superior frontal gyrus, **IFG**: inferior frontal gyrus, **tri**: triangularis, **oper**: opercularis.

S2.4 Connectivity change - behavior change

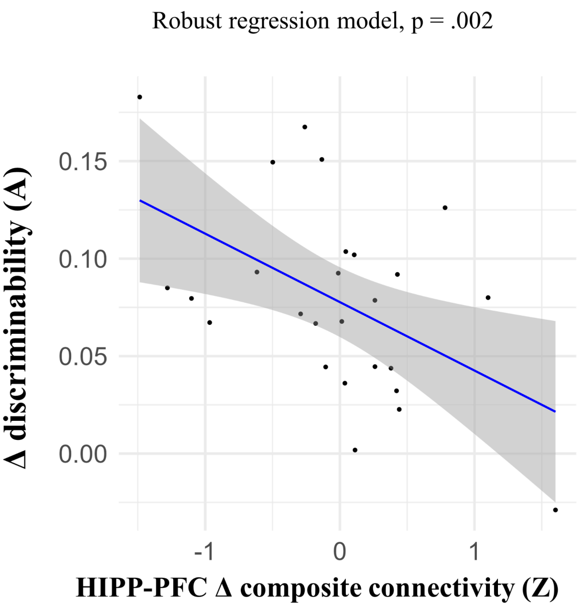

Figure S4. The hippocampal-PFC connectivity decrease-MD improvement association is replicated using robust regression (Δ: post- minus pre-training). Robust linear regression model fitted within the training group (n=26). X axis depicts the independent variable: LD connectivity Δ values (gPPI) of a connection (seed ROI – target ROI) or a composite score of a group of connections. Y axis shows the dependent variable (discriminability index A). The p value for the robust model is shown. **HIPP-PFC**: hippocampal-prefrontal connectivity composite score (Z score).

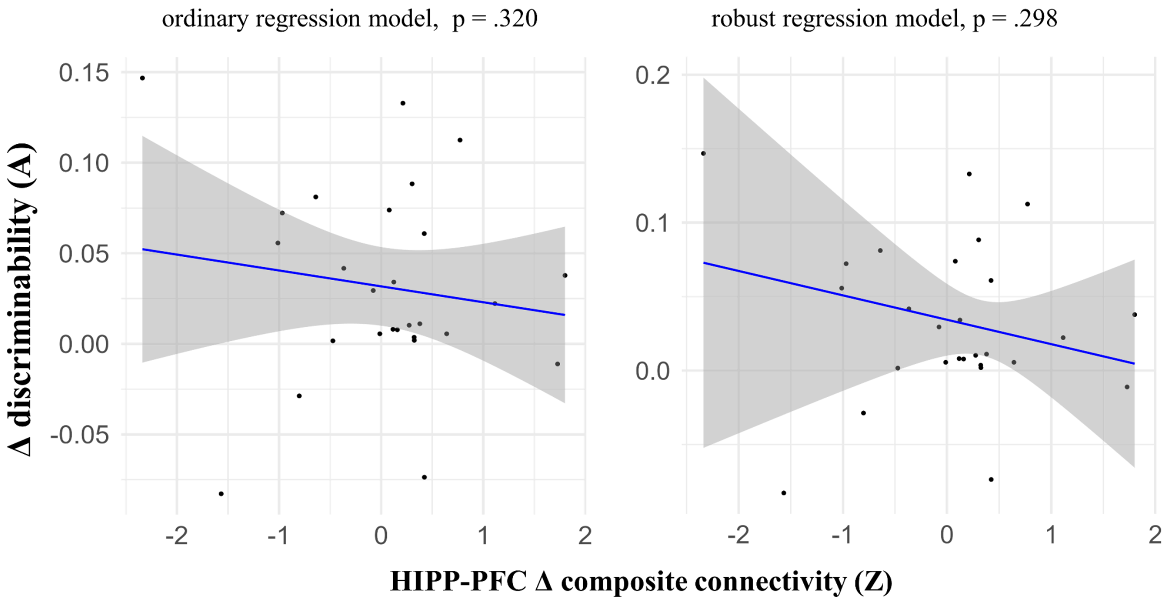

Figure S5. There is no connectivity change associated with MD improvement in the control group (Δ: post- minus pre-training change). Regular and robust linear regression model fitted within the control group (n=27). X axis depicts the independent variable: LD connectivity Δ values (gPPI) of the composite score of the hippocampal-PFC group of connections. Y axis shows the dependent variable (discriminability index A). The p value in each model is shown. **HIPP-PFC**: hippocampal-prefrontal connectivity composite score (Z score).

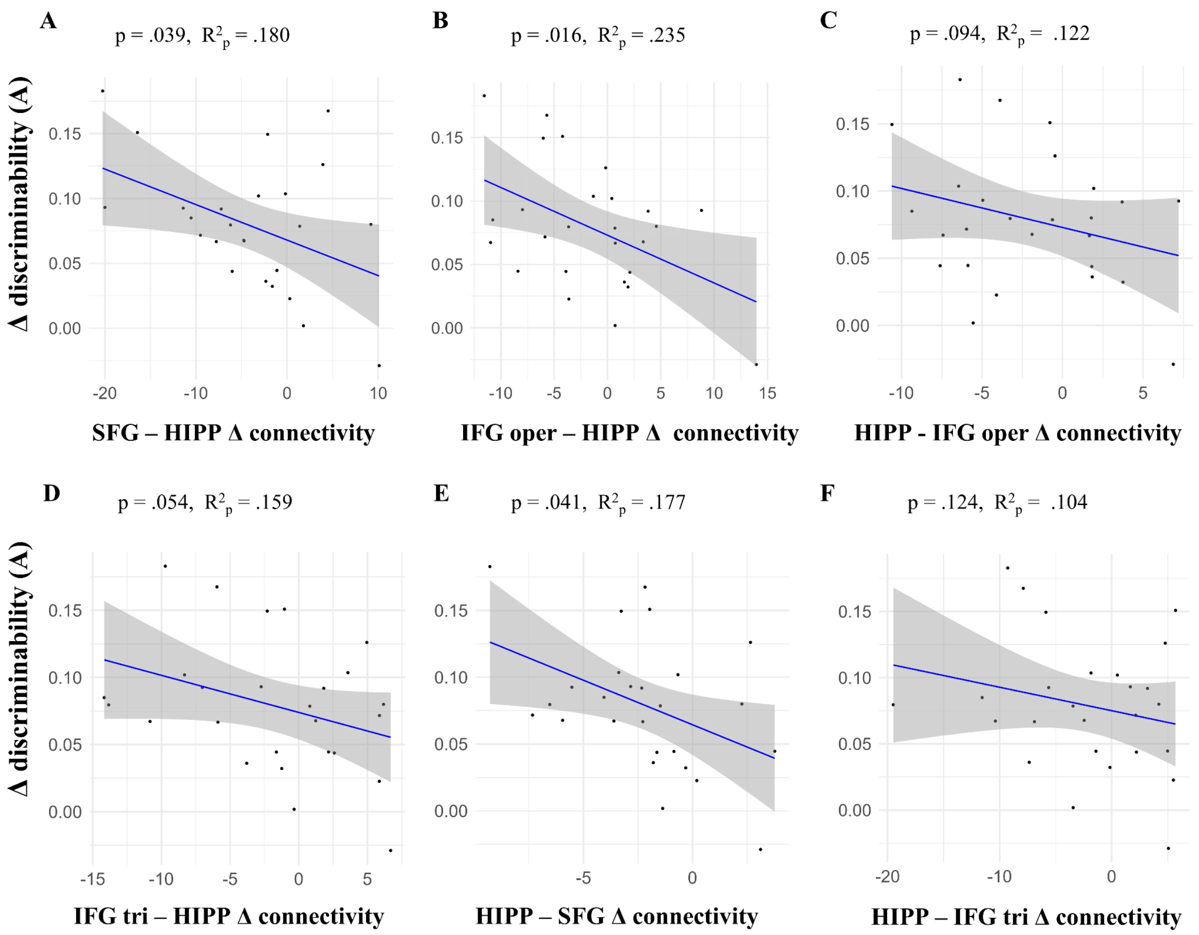

Figure S6. **Connectivity change-behavior change models fitted separately for each hippocampal-PFC connection**. A linear regression model was fitted for each connection (covariates: age, gender). The X axis depicts the independent variable: LD connectivity change (Δ: post- minus pre-training) values (gPPI) in a certain connection (seed ROI – target ROI), here an individual hippocampal-PFC connection (as those were identified at the cluster 3 of the baseline-analysis). The Y axis shows the dependent variable: A discriminability change. The p value and partial R square values are shown. In the connections depicted (A-F) we observe a pattern of a negative link with lower connectivity being associated with higher MD performance change. No FDR correction is applied here since this analysis was conducted as a clarification follow-up to our main analysis (which focused on the composite connectivity score).

HIPP: hippocampus**, SFG**: superior frontal gyrus, **IFG**: inferior frontal gyrus, **tri**: triangularis, **oper**: opercularis.

S2.5 Comparison of motion for group x time point for the functional data.

A mixed-design ANOVA was performed to assess differences in mean framewise displacement (FD) between groups (training vs. control) and across time points (pre- vs. post-training), with age and gender included as covariates. The analysis revealed no

significant main effects of group (F(1,49) = 0.03, p = .865), time point (F(1,49) = 1.63, p = .208), or their interaction (F(1,49) = 1.07, p = .307) on motion. These results indicate that head motion was comparable between groups and across sessions. See Supplementary Figure S7 for the distribution of mean FD values by group and time point.

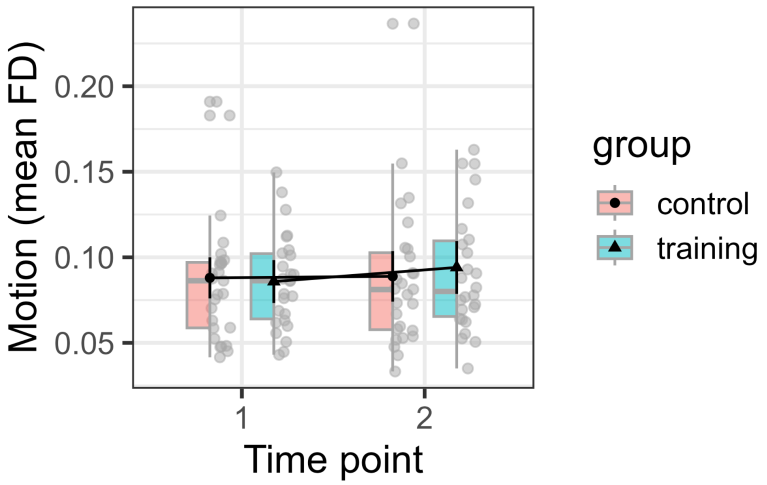

Figure S7. No motion differences for the functional imaging data between the groups. Motion values for the functional imaging data plotted by group x time point. The X axis depicts the independent variable time point (1= pre, 2 = post), while the Y axis depicts the mean framewise displacement (FD) value per subject which denotes motion. There are no systematic differences in terms of motion between the groups (training versus control) and time point (pre- versus post- training).

Boxes show the median (center line) and interquartile range (IQR); whiskers extend to 1.5×IQR; gray points are individual participants. Overlaid black markers/lines are marginal means with 95% confidence intervals (control n = 27, training n = 26).

S2.6 Condition-specific decomposition of the LD effect: Correct-lures drive the connectivity changes

To test whether the post-training LOC–OP LD effect (Fig. 6) is driven by correct-lure rather than the repeat trials, we computed condition-specific gPPI changes (post − pre) for the correct lures (CL) vs baseline and repeats (Reps) vs baseline. Each condition reflected task-modulated connectivity relative to the perceptual baseline trials. We compared these conditions within the training group (n=26) by performing a within-samples t-test. The within-subjects difference of changes was significant (Fig. S8) (ΔCL − ΔReps = 6.82, t(25) = 4.62, p = .0001) indicating that the LD connectivity change we found is attributable to the

correct-lure condition. Consistent with this, the connectivity change for repeats alone did not differ from zero (mean ΔReps = −0.31, $t(25) = -0.17$, $p = .863$).

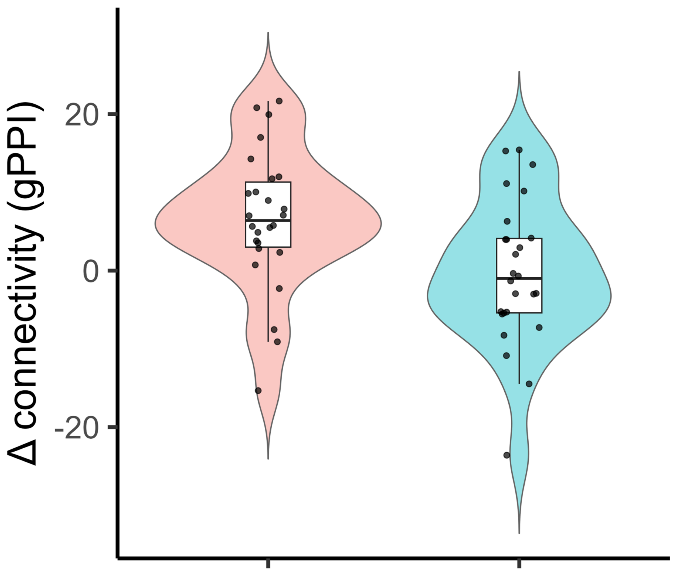

Figure S8. The LOC–OP post-training connectivity change is driven by the correct-lures condition. The plot shows within-subject gPPI connectivity change (Δ = post − pre), within the cognitive training group (n=26), for the following two conditions: correct lures vs baseline and repeats vs baseline (i.e. task-modulated connectivity within each condition relative to the perceptual baseline trials). The within-subjects difference of changes was significant ($p = .0001$), indicating that the LD (correct lures − repeats) change is driven by changes during the correct-lures. The repeats' connectivity change alone did not differ from zero ($p = 0.863$). Boxes show the median (center line) and interquartile range, black lines show the plots' whiskers.

S2.7 The connectivity decrease-MD improvement association is replicated using the correct lures minus false-alarms contrast

To confirm the robustness of the association between post-training hippocampal-PFC connectivity decrease and MD improvement (see Fig. 7), we performed a sensitivity analysis using the correct minus false-alarms (incorrect lures) fMRI contrast. We fitted a change–change linear regression model within each group (Δ connectivity → Δ MD performance, covariates: age, sex). Consistent with our primary results using the LD contrast, we found a significant negative association in the training group, where greater decreases in correct-incorrect lures connectivity were associated with larger improvements in MD (p =

.012, partial R^2 = .252; Fig. S9A). This association was not significant in the control group (p = .327, partial R^2 = .042; Fig. S9B). These results demonstrate that the training-linked connectivity-behavior relationship is robust across different contrasts.

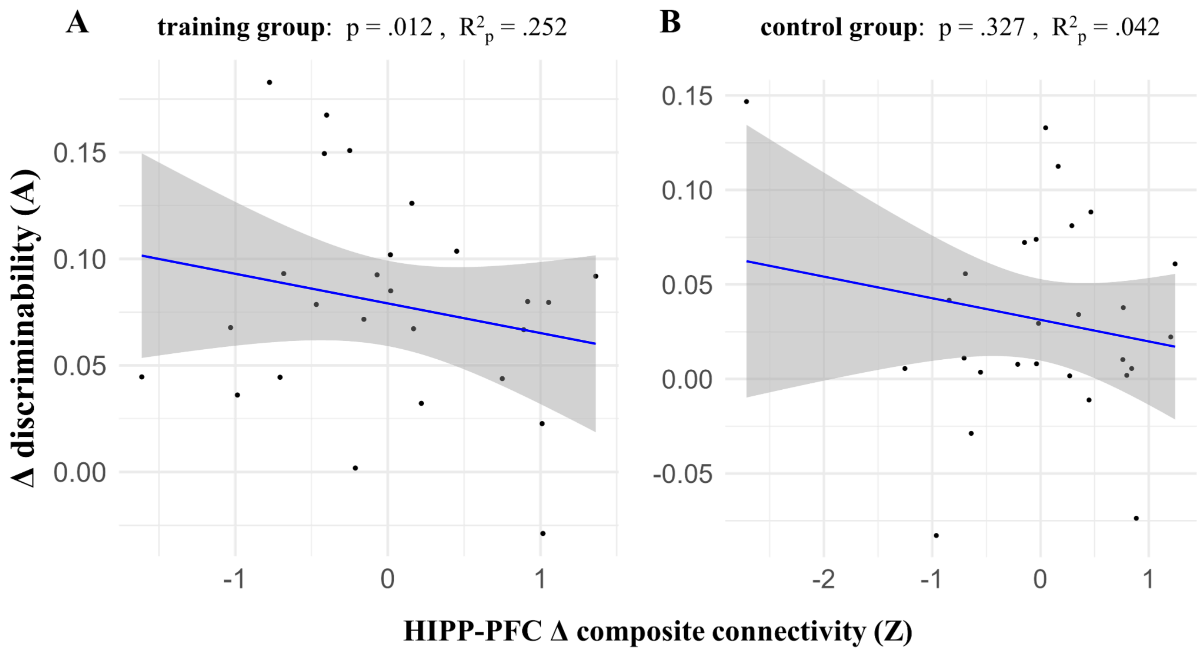

Figure S9. Hippocampal-PFC connectivity change–MD improvement association using the correct lure-incorrect lure fMRI contrast. Scatterplots show the relationship between the post-training change (Δ) in hippocampal–PFC composite connectivity (z-score) and in MD performance (discriminability A′), separately for the (A) training group and (B) control group. In the training group, a significant negative association was found (p = .012, partial R^2 = .252), such that larger decreases in correct-incorrect lures connectivity relate to larger MD gains. This association was not significant in the control group (p = .327, partial R^2 = .042). Δ represents the post- minus pre-training values.

Abbreviations in the manuscript

AD: Alzheimer's disorder

CL: correct lures

Δ: Delta (change post versus pre-training)

fMRI: functional magnetic resonance imaging

gPPI: generalized psychophysiological interaction

HIPP: hippocampus

IFG: inferior frontal gyrus; tri: triangularis, oper: opercularis

LD: lure detection

LDI: lure discrimination index

LOC: lateral occipital cortex

MD: mnemonic discrimination

MedFC: medial frontal cortex

MidFG: middle frontal gyrus

MTL: medial temporal lobe

MST: mnemonic similarity task

OP: occipital pole

ORR: object-in-room recall

PaHC: parahippocampal cortex

PFC: prefrontal cortex

PRC: perirhinal cortex

Reps: repeats

ROI: region of interest

ROCF: Rey-Osterrieth Complex Figure

SFG: superior frontal gyrus

VLMT: verbal learning and memory test
