# Supplementary material for: Hippocampal-prefrontal connectivity relates to inter-individual differences and training gains in distinguishing similar memories
Source: Commun Biol. 2025 Dec 28;9:129. doi: 10.1038/s42003-025-09408-7 (PMC12855975; doi:10.1038/s42003-025-09408-7)
Supplement: Supplementary file 3 — Description of Additional Supplementary Files [file 42003_2025_9408_MOESM3_ESM.pdf]

Description of Additional Supplementary Files:

File: Supplementary Data 1

Description: Source Data for figures

File: Supplementary Data 2

Description: Source Data for tables
