# Supplementary material for: Hippocampal-prefrontal connectivity relates to inter-individual differences and training gains in distinguishing similar memories
Source: Commun Biol. 2025 Dec 28;9:129. doi: 10.1038/s42003-025-09408-7 (PMC12855975; doi:10.1038/s42003-025-09408-7)
Supplement: Supplementary file 6 — Reporting Summary [file 42003_2025_9408_MOESM6_ESM.pdf]

nature portfolio

Corresponding author(s): Panagiotis Iliopoulos

Last updated by author(s): Nov 20, 2025

Reporting Summary

Nature Portfolio wishes to improve the reproducibility of the work that we publish. This form provides structure for consistency and transparency in reporting. For further information on Nature Portfolio policies, see our Editorial Policies and the Editorial Policy Checklist.

Statistics

For all statistical analyses, confirm that the following items are present in the figure legend, table legend, main text, or Methods section.

n/a	Confirmed	
☐	☒	The exact sample size (n) for each experimental group/condition, given as a discrete number and unit of measurement
☐	☒	A statement on whether measurements were taken from distinct samples or whether the same sample was measured repeatedly
☐	☒	The statistical test(s) used AND whether they are one- or two-sided<br>*Only common tests should be described solely by name; describe more complex techniques in the Methods section.*
☐	☒	A description of all covariates tested
☐	☒	A description of any assumptions or corrections, such as tests of normality and adjustment for multiple comparisons
☐	☒	A full description of the statistical parameters including central tendency (e.g. means) or other basic estimates (e.g. regression coefficient) AND variation (e.g. standard deviation) or associated estimates of uncertainty (e.g. confidence intervals)
☐	☒	For null hypothesis testing, the test statistic (e.g. F, t, r) with confidence intervals, effect sizes, degrees of freedom and P value noted<br>*Give P values as exact values whenever suitable.*
☐	☒	For Bayesian analysis, information on the choice of priors and Markov chain Monte Carlo settings
☐	☒	For hierarchical and complex designs, identification of the appropriate level for tests and full reporting of outcomes
☐	☒	Estimates of effect sizes (e.g. Cohen's d, Pearson's r), indicating how they were calculated

Our web collection on statistics for biologists contains articles on many of the points above.

Software and code

Policy information about availability of computer code

Data collection	The "Presentation" software for experimental design was used for the fMRI experimental task and the behavioral tasks, as well as the custom code provided by the Stark Lab for the MST (Mnemonic similarity task). Specifically, the MST (Version 0.9) (Stark et al., 2013) was used which assesses mnemonic discrimination through separate encoding and retrieval phases
Data analysis	To analyze the data we used: MATLAB custom scripts to handle and prepare the fMRI data and the CONN Toolbox (version 21a.) to analyze the fMRI data, the R statistical language (R version 4.4.2) to run behavior-connectivity relationships and behavioral analysis, the 'fMRIPrep' (v. 20.2.6) pipeline was used to preprocess the fMRI data.

For manuscripts utilizing custom algorithms or software that are central to the research but not yet described in published literature, software must be made available to editors and reviewers. We strongly encourage code deposition in a community repository (e.g. GitHub). See the Nature Portfolio guidelines for submitting code & software for further information.

Data

Policy information about availability of data

All manuscripts must include a data availability statement. This statement should provide the following information, where applicable:

- Accession codes, unique identifiers, or web links for publicly available datasets
- A description of any restrictions on data availability
- For clinical datasets or third party data, please ensure that the statement adheres to our policy

The data employed in this study are not publicly available.

Research involving human participants, their data, or biological material

Policy information about studies with human participants or human data. See also policy information about sex, gender (identity/presentation), and sexual orientation and race, ethnicity and racism.

Reporting on sex and gender	Gender was used as covariate in our analyses. The sample characteristics are as following: Sixty young adults were recruited. Five participants voluntarily chose to discontinue with the study and one was excluded due to problematic brain extraction (M age = 23.76 years, SD = 3.35, 61.11% female).
Reporting on race, ethnicity, or other socially relevant groupings	All participants were young adults german speakers, not race variability is reported.
Population characteristics	Sixty young adults were recruited. Five participants voluntarily chose to discontinue with the study and one was excluded due to problematic brain extraction (M age = 23.76 years, SD = 3.35, 61.11% female).
Recruitment	Participants were recruited via advertising, participants were young adults university students. There was no systematic bias in their selection.
Ethics oversight	The study was approved by the Otto von Guericke University Magdeburg ethics committee and conducted in accordance with the declaration of Helsinki, 2013.

Note that full information on the approval of the study protocol must also be provided in the manuscript.

Field-specific reporting

Please select the one below that is the best fit for your research. If you are not sure, read the appropriate sections before making your selection.

☐ Life sciences ☒ Behavioural & social sciences ☐ Ecological, evolutionary & environmental sciences

For a reference copy of the document with all sections, see nature.com/documents/nr-reporting-summary-flat.pdf

Behavioural & social sciences study design

All studies must disclose on these points even when the disclosure is negative.

Study description	To assess the impact of cognitive training on mnemonic discrimination (MD) and its neural mechanisms, we analyzed behavioral and fMRI data from 54 healthy young adults who completed pre- and post-intervention sessions. Participants were randomly assigned to either the experimental training group (n=26), which received MD-focused cognitive training, or the active control group (n=27), which engaged in a psychomotor task without an MD component (Fig.1)<br><br>During fMRI scanning sessions, all participants performed a 6-back object-scene recognition task that required distinguishing between highly similar ("lure") and identical ("old") images, engaging MD (Fig.1A-C). Detailed descriptions of the participant groups, behavioral tasks and training protocol are provided in the Methods section and in (Güsten et al., 2024). For an overview of the analysis flow see Fig. 2.
Research sample	Participants were recruited via advertising, participants were young adults university students. Sixty young adults were recruited. Five participants voluntarily chose to discontinue with the study and one was excluded due to problematic brain extraction (M age = 23.76 years, SD = 3.35, 61.11% female).
Sampling strategy	Random sampling strategy was used. We have a sample size which is in the standard range of fMRI experimental studies (~ 60 subjects). Since this was an interventational study with two time points, there were approximately 60 x 2 scanning sessions.
Data collection	Pre- and post-training, participants performed at the scanner, while fMRI data was collected, a 6-back version of the object-scene task developed to measure MD (Berron et al., 2018; Güsten et al., 2021) (see Fig. 1D). Before scanning, subjects were given standardized instructions and underwent a short training session. Stimuli were presented via a mirror on a magnetic resonance (MR) compatible display. When necessary, vision was corrected using MR-compatible glasses.

Computer software was used for most tasks, as well as paper and pencil for the: modified 30-word list verbal learning memory task (Helmstaedter et al., 2001), and the Rey-Osterrieth Complex Figure (ROCF) (Osterrieth, 1944).

Participants performed a 2-week computerized MD task training on a newly programmed online platform (http://iknd-games.ovgu.de/MemTrain/), comprising 6 training sessions in total. Each training session lasted 45 minutes, in addition to breaks between training runs.

Timing: August 2018 till March 2020

Data exclusions: One subject was excluded from the MRI analyses due to problematic brain extraction (fMRIprep pipeline)

Non-participation: Five participants voluntarily chose to discontinue with the study.

Randomization: Allocation to groups was random.

Reporting for specific materials, systems and methods

We require information from authors about some types of materials, experimental systems and methods used in many studies. Here, indicate whether each material, system or method listed is relevant to your study. If you are not sure if a list item applies to your research, read the appropriate section before selecting a response.

Materials & experimental systems

n/a	Involved in the study
☒	☐ Antibodies
☒	☐ Eukaryotic cell lines
☒	☐ Palaeontology and archaeology
☒	☐ Animals and other organisms
☒	☐ Clinical data
☒	☐ Dual use research of concern
☒	☐ Plants

Methods

n/a	Involved in the study
☒	☐ ChIP-seq
☒	☐ Flow cytometry
☐	☒ MRI-based neuroimaging

Plants

Seed stocks: *Report on the source of all seed stocks or other plant material used. If applicable, state the seed stock centre and catalogue number. If plant specimens were collected from the field, describe the collection location, date and sampling procedures.*

Novel plant genotypes: *Describe the methods by which all novel plant genotypes were produced. This includes those generated by transgenic approaches, gene editing, chemical/radiation-based mutagenesis and hybridization. For transgenic lines, describe the transformation method, the number of independent lines analyzed and the generation upon which experiments were performed. For gene-edited lines, describe the editor used, the endogenous sequence targeted for editing, the targeting guide RNA sequence (if applicable) and how the editor was applied.*

Authentication: *Describe any authentication procedures for each seed stock used or novel genotype generated. Describe any experiments used to assess the effect of a mutation and, where applicable, how potential secondary effects (e.g. second site T-DNA insertions, mosiacism, off-target gene editing) were examined.*

Magnetic resonance imaging

Experimental design

Design type: task event-related design

Design specifications: The fMRI task had 2 x 13 mins runs. Stimuli were presented in sequences of 12 items each
In total, 60 sequences were shown. This resulted in a 2x2 factorial design with 30 trials for each trial type combination (lure objects, repeats object, lure scenes, repeats scenes). The stimuli were presented for 3 seconds each. Jittered inter-stimulus intervals were used (range: 0.8 to 4.2 secs, mean ~ 1.63 secs).

Behavioral performance measures: Correct button responses (accuracy) were recorded. The mean and standard deviation across subjects were calculated.

Acquisition

Imaging type(s) | functional MRI (fMRI)

Field strength | 3 Tesla

Sequence & imaging parameters | Whole-brain functional data were acquired using T2*-weighted echo planar imaging (EPI). We used a 2D simultaneous multi-slice EPI sequence (SMS-EPI), with a 2 x 2 mm² resolution, FOV = 212 x 212mm², TR/TE = 2200/30ms, 10% slice gap, multiband acceleration factor 2, GRAPPA 2, phase encoding (PE) direction P > A, 64 slices and 2mm slice thickness. In addition, phase maps were acquired with the following parameters: TR/TE1/TE2 = 675/4.92/7.38 ms, spatial resolution = 3 mm, and 48 slices.

Area of acquisition | Whole brain scan

Diffusion MRI ☐ Used ☒ Not used

Preprocessing

Preprocessing software | 'fMRIPrep' (v. 20.2.6) standard default pipeline and Conn Matlab Toolbox (version 21a). The functional data were smoothed using a 4 mm FWHM Gaussian kernel (determined a-priori as twice the voxel size in order to compromise anatomical specificity and signal-to-noise ratio increase). However, the smoothing kernel size is not applicable for our ROI analysis, since CONN uses the unsmoothed ROI data. Each T1-w image was corrected for intensity non-uniformity and skull-stripped. A T1w-reference map was computed by registering 2 T1w images acquired from each subject.

Normalization | Spatial normalization to the MNI space was performed through nonlinear registration. The functional data were slice-time corrected and co-registered to the T1w using boundary-based registration with 9 degrees of freedom. All resampling was performed in a single interpolation step.

Normalization template | MNI space (ICBM 152 Nonlinear Asymmetrical template 2009c)

Noise and artifact removal | Physiological noise regressors were extracted using CompCor, in addition to other confound variables, including head-motion parameters and framewise displacement (FD), which were used later at denoising. Frames that exceeded a threshold 0.5 mm FD were annotated as motion outliers.
The denoising steps included: regressing out 3 translation and 3 rotation motion parameters plus their first-order derivatives (12 parameters in total), anatomical component-based noise correction procedure (aCompCor) to remove noise components from the white matter and cerebrospinal areas, scrubbing to remove the volumes identified as outliers due to excessive motion (> 0.5 mm FD), high-pass filtering 1/128 Hz, linear detrending, and despiking. Covariates were also included to account for potential slow trends, initial magnetization transients, or constant task-induced responses in the BOLD signal, according to the default denoising pipeline in CONN.

Volume censoring | scrubbing to remove the volumes identified as outliers due to excessive motion (> 0.5 mm FD)

Statistical modeling & inference

Model type and settings | We applied a region of interest generalized psychophysiological interaction (gPPI) analysis (McLaren et al., 2012) to examine the task-modulated functional connectivity between the ROIs in our model (ROI-to-ROI analyses). We performed hypothesis-driven region of interest (ROI)-to-ROI analysis. We constructed a model including major medial temporal lobe (MTL), prefrontal (PFC) and visual ROIs (Fig. 2).
First-model was based on fixed effects and second-level on random effects according to standard practices in the CONN toolbox and use of gPPI for event-related designs. Furthermore, we used linear regression models to test the relationship of connectivity and memory performance

Effect(s) tested | We tested for fMRI connectivity effects in the successful MD contrast (lure detection: correct lure minus repeat trials). All functional connectivity analyses performed in our study refer to this LD contrast. F statistics were obtained for each cluster, with T values calculated for each connection belonging to each cluster (see Table 1).

gPPI values were calculated for each ROI-to-ROI connection pair for each participant, representing lure detection (LD) task functional connectivity from a seed to a target region. gPPI measures the influence of a seed on a target region after partialling out task-related activity and task-unrelated connectivity, resulting in an asymmetrical effective connectivity matrix. This means that each connection given follows a seed – target format that denotes directionality.

In a later step, the training-related interaction: experimental Group x Time (pre,post) was also calculated for the brain connections of each cluster using ANOVA, as well as for the behavioral mnemonic discrimination data (A discriminability index).
Furhermore, linear regressions models were fitted to test the relationship of brain connectivity and memory performance at baseline (pre-training), as well as between the brain connectivity change and memory performance change (change-change relationship).

Specify type of analysis: ☐ Whole brain ☒ ROI-based ☐ Both

Anatomical location(s)	hippocampus, enthorhinal cortex (EC), perirhinal cortex (PRC), parahippocampal cortex (PaHC), superior frontal gyrus (SFG), middle frontal gyrus (MidFG), and inferior frontal gyrus (IFG) including pars triangularis (tri) and pars opercularis (par), medial prefrontal cortex (medFC), lateral occipital cortex (LOC) and occipital pole (OP) (Fig. 2)

Statistic type for inference (See Eklund et al. 2016)	cluster-level inference with the default ROI-to-ROI network multivariate parametric statistics approach in CONN. This approach uses multivariate statistics to examine groups of related connections, resulting in an F-statistic for each cluster with FDR-corrected cluster-level p-values, in addition to a post-hoc connection-level thresholding that keeps the strongest connections within each significant cluster. We used the standard settings: $p < .05$ cluster-level p-FDR, connection threshold: $p < .05$ p-uncorrected.
Correction	FDR cluster-level correction. FDR correction was also used for the linear regressions models which tested the relationship between connectivity and behavior (mnemonic discrimination performance)

Models & analysis

n/a	Involved in the study
☐	☒ Functional and/or effective connectivity
☒	☐ Graph analysis
☒	☐ Multivariate modeling or predictive analysis

Functional and/or effective connectivity	A generalized psychophysiological interaction (gPPI) analysis was used to model event-related task connectivity. No other measure was used to measure connectivity.
